# Effect of Transcutol P on Percutaneous Absorption of Gel Containing Pure Synthetic Caffeine or Plant‐Extracted Natural Caffeine

**DOI:** 10.1155/drp/3538935

**Published:** 2026-08-07

**Authors:** Nastaran Seyyed Abbasi, Fatemeh Soltanmohammadi, Mohammad Charkhpour, Javad Shokri

**Affiliations:** ^1^ Student Research Committee, Tabriz University of Medical Sciences, Tabriz, Iran, tbzmed.ac.ir; ^2^ Department of Pharmaceutics, Faculty of Pharmacy, Tabriz University of Medical Sciences, Tabriz, Iran, tbzmed.ac.ir; ^3^ Department of Pharmacology, Faculty of Pharmacy, Tabriz University of Medical Sciences, Tabriz, Iran, tbzmed.ac.ir

**Keywords:** caffeine, cellulite, skin, skin permeability, Transcutol

## Abstract

**Background:**

Caffeine, a safe methylxanthine, has been widely used for the treatment of skin diseases such as cellulite, hair loss, aging, and psoriasis. However, its skin penetration is low. Transcutol is a biocompatible, nonvolatile permeation enhancer which solubilize large number of drugs and does not change the integrity of the skin structure.

**Materials and methods:**

In this study, the skin permeability of synthetic and natural caffeine gels was compared by ex vivo experiments. Furthermore, Transcutol P was used as a permeation enhancer, and its impact on skin permeation enhancement was determined.

**Results:**

The skin penetration of caffeine from the formulation containing coffee extract, a type of natural caffeine, is higher than that of the formulation containing pure synthetic caffeine. This effect is likely due to the presence of coffee, which can act as a skin permeation enhancer. Additionally, the use of Transcutol P in a formulation containing coffee extract at a concentration of 2.5% caused the highest amount of flux. Therefore, the formulation of gel containing natural caffeine along with 2.5% Transcutol is optimal and can be used for further studies.

**Conclusion:**

It can be concluded that the addition of 2.5% Transcutol P in the formulation containing coffee extract resulted in the highest skin permeation.

## 1. Introduction

Methylxanthines such as caffeine, aminophylline, and theophylline have been used for the treatment of asthma, neurodegenerative diseases, acute exacerbation of chronic obstructive pulmonary disease, peripheral vascular disease, etc. [1–5]. Coffee, tea, and chocolate are natural sources of caffeine [6]. Extracting caffeine from natural sources is more expensive than using synthetic caffeine. Despite this cost difference, natural caffeine contains other beneficial compounds such as antioxidants, which have been linked to positive health outcomes [7, 8]. Caffeine, which is considered safer than both aminophylline and theophylline [9], inhibits the activation of phosphodiesterase (PDE), leading to lipolysis. This inhibition increases the levels of cyclic adenosine monophosphate (cAMP), which in turn stimulates hormone‐sensitive lipase and reduces cellulite [10]. Caffeine not only inhibits PDE but also activates *β*‐adrenergic receptors, which further increases cAMP levels and activates hormone‐sensitive lipase [11]. This alkaloid also blocks *α*‐adrenergic receptors, preventing excessive fat accumulation and promoting lipolysis [10, 12]. Additionally, caffeine improves local blood circulation and has antioxidant properties [10]. Caffeine also, can protect the skin against UV irradiation [13]. Topical caffeine can promote human hair follicle growth and decrease Psoriatic Area and Severity Index (PASI) in patients with psoriasis vulgaris [14, 15]. Previous studies have shown that caffeine has low skin permeability due to its low logP (−0.07), resulting in most of the applied caffeine remaining unabsorbed on the skin [16]. One way to address this issue is by using skin permeation enhancers, which can decrease the skin’s barrier properties or increase the amount and rate of drug transmission through the stratum corneum [17, 18]. These enhancers work by disorganizing and disrupting stratum corneum lipids, interacting with intracellular proteins, improving skin hydration, or enhancing partitioning in the stratum corneum [19, 20]. One such enhancer is Transcutol P, a highly purified form of diethylene glycol monoethyl ether solvent. This hydro alcoholic solvent has properties such as biocompatibility, the ability to solubilize a large number of drugs, nonvolatility, and the ability to maintain the integrity of the skin structure, making it an ideal penetration enhancer [20, 21].

Despite previous reports on the permeation‐enhancing effect of Transcutol P on synthetic caffeine formulations [22], limited information is available regarding its effect on formulations prepared from natural caffeine sources such as *Coffea canephora* extract. Therefore, the present study aimed to compare the ex vivo skin permeation of gels containing synthetic caffeine and naturally derived caffeine from *C. canephora* extract. In addition, the effect of Transcutol P as a penetration enhancer on caffeine permeation was investigated. It was hypothesized that the natural caffeine formulation, due to the presence of additional phytochemical constituents in the extract, may exhibit different permeation behavior compared with synthetic caffeine formulations.

## 2. Materials and Methods

### 2.1. Materials and Chemicals

Caffeine powder, trimethylamine, sodium dihydrogen phosphate, sodium hydroxide, propylene glycol (PG), propanol, ethanol, acetone, triethanolamine, and methanol were purchased from Merck (Darmstadt, Germany). Potassium dihydrogen phosphate (AppliChem, USA), Carbopol 934 (Sigma Aldrich, Dorset, UK), normal saline serum (Shahid Ghazi, Tabriz, Iran), and Transcutol P (Sigma Aldrich, Dorset, UK) were obtained.

### 2.2. *C. canephora* Extract Preparation

*C. canephora* beans were ground using a laboratory mill (IKA® A11 basic analytical mill) to a fine powder. The particle size of the powder was not sieved but was consistently produced by a 30‐s milling cycle. Next, 500 g of the resulting powder was soaked in 1.2 L of a 70:30 v/v water:ethanol solvent at 40°C for 3 days with periodic stirring (every 12 h for 10 min at 200 rpm using a magnetic stirrer). After each stirring, the solvent was refreshed. The combined extracts were then filtered (Whatman No. 1 filter paper), and the solvent was removed using a rotary evaporator (Heidolph Hei‐VAP Advantage, Germany) at 40°C under reduced pressure (175 mbar) to obtain a dry extract. The concentration of caffeine in the final extract was determined using the HPLC method described in Section 2.4.

### 2.3. Determination of Maximum Absorption Wavelength by UV Spectrophotometry

The maximum UV absorption (λmax) for a 10 ppm solution of synthetic caffeine powder in the HPLC mobile phase was determined by scanning from 200 to 400 nm using a UV–Vis spectrophotometer (Shimadzu UV‐1800, Japan). The highest absorption was confirmed to be at a wavelength of 273 nm, which was subsequently used for HPLC detection.

### 2.4. Determination of Caffeine Concentration by UV–Visible Spectrophotometer

The concentration of caffeine in the *C. canephora* extract was quantified using an HPLC‐UV system. The HPLC system (Knauer, Germany) was equipped with a Pump 1000, a UV Detector 2500, and an Autosampler 3950. Separation was achieved using a KNAUER Eurospher II C18 column (4.6 × 250 mm, 5‐μm particle size) maintained at 40°C. The isocratic mobile phase consisted of a water/methanol mixture (75:25, v/v), filtered through a 0.45‐μm nylon membrane and degassed by sonication for 15 min prior to use. The flow rate was 0.8 mL/min, the injection volume was 30 μL, and the UV detector was set to 273 nm. The total run time was 10 min.

A synthetic caffeine stock solution (1000 ppm) was prepared in methanol. This was serially diluted with the mobile phase to create eight working standard solutions with concentrations of 0.625, 1.25, 2.5, 5, 10, 20, 40, and 80 ppm. Each standard was injected in triplicate. The calibration curve was constructed by plotting the mean peak area against the concentration. The curve demonstrated excellent linearity over the range of 0.625–80 ppm with a correlation coefficient (*R* ^2^) of > 0.999.

For the extract analysis, a stock solution of the dry *C. canephora* extract was prepared in methanol at a concentration of approximately 10 mg/mL and then diluted appropriately with the mobile phase to fall within the linear range of the calibration curve. The sample was filtered through a 0.22‐μm syringe filter before injection. The concentration of caffeine in the extract was calculated by comparing the peak area of the sample to the calibration curve. The results indicated that the caffeine content in the dry extract was 10% w/w (e.g., the extract was 10% caffeine by weight, meaning 0.1 g caffeine per 1 g of extract). The caffeine peak in both standard and sample chromatograms had a retention time of approximately 6.78 min.

### 2.5. Preparation Gel of Synthetic/Natural Caffeine Gel

A 0.5% w/w Carbopol gel base was prepared first. Specifically, 0.5 g of Carbopol 934 was slowly dispersed in 30 mL of deionized water under moderate magnetic stirring (500 rpm) and allowed to hydrate for 1 h.

For the synthetic caffeine gel, 0.5 g of synthetic caffeine was dissolved in 31.25 mL of 90% (w/w) ethanol. This solution was chosen to ensure complete dissolution of the drug. This solution was then incorporated into the Carbopol dispersion. Subsequently, 10 mL of PG was added as a penetration enhancer and humectant. The mixture was stirred continuously, and the volume was adjusted to 100 mL with deionized water. Finally, the gel was neutralized to a pH of 5.5–6.0 by adding approximately 0.5 mL of triethanolamine (10% w/w) dropwise to form the gel matrix.

For the natural caffeine gel, an equivalent amount of *C. canephora* extract powder containing 0.5 g of caffeine was used. Based on the HPLC quantification (Section 2.4), this required 5 g of the extract. Thus, both the synthetic caffeine gel and the coffee‐extract gel were formulated to contain the same final caffeine concentration (0.5% w/w), allowing direct comparison between formulations. The same procedure as for the synthetic gel was followed.

All freshly prepared gels were visually inspected for homogeneity and then stored at room temperature for 24 h. Only gels that remained clear, homogeneous, and without phase separation or syneresis after this period were deemed stable and selected for further experiments.

### 2.6. Ex Vivo Skin Permeation Study

Male Wistar rats weighing 150–180 g were euthanized by intraperitoneal administration of sodium pentobarbital at a dose of 150 mg/kg body weight. Following confirmation of death, the abdominal hair was removed using an electric razor, and the abdominal skin was excised. The experiments were performed in accordance with the guidelines of the Ethics Committee of Tabriz University of Medical Sciences (Approval No. IR.TBZMED.VCR.REC.1400.083).

The abdominal skin was carefully separated, and the subcutaneous fat was removed. Before starting the diffusion experiment, the dermal side of the skin was kept in contact with normal saline solution and refrigerated at 4°C for up to 4 h to remove extraneous debris and allow hydration of the tissue. Franz‐type diffusion cells (Erweka HDT6, Germany) with an effective diffusion area of 3.8 cm^2^ and a receptor volume of 26 cm^3^ were used for the skin permeation studies.

Six Franz cells were located on the skin penetration study apparatus. Each set of investigations was done with a minimum of 3 diffusion cells (*n* ≥ 3) and repeated 3 times on different days. The receptor compartment was filled with 26 mL of PBS (pH 7.4). A precisely weighed amount of 1.0 g (±0.01 g) of the prepared caffeine gel was applied to the center of the skin surface in the donor compartment. The gel was then spread evenly across the entire 3.8‐cm^2^ diffusion area using a flat‐tipped micro‐spatula to ensure a uniform layer without air pockets. The receptor medium temperature was maintained at 37°C, and it was stirred at 500 rpm with magnetic stirrers. The receptor chamber was maintained at 37°C. This standard setup, validated in previous studies [23], ensures that the skin surface temperature is maintained at approximately 32°C, mimicking physiological conditions. 500 μL samples were collected from the receptor phase at determined time intervals (0, 5, 10, 15, 30, 45, 60, and 90 min) and replaced with the same volume of PBS at 37°C to keep the volume constant. The amount of caffeine was determined by HPLC.

As the study was conducted with rat skin, caution should be taken in interpreting the results in relation to the human situation. It is well known that the rat skin barrier is much more permeable than the human skin barrier [24, 25], and more importantly, the passage routes may vary.

Rat skin is significantly different in structure and barrier as compared to human skin. This is related to the situation that the rat is a hairy animal with many more hair follicles per skin area and a thinner stratum corneum. By this, the effect of bypassing the stratum corneum barrier by diffusion via transappendigial routes may be a relevant confounding factor in rats that will not become that effective in humans. As long as the relation of possible routes of exposure, intracellular, intercellular, and transappendigial is not taken into account, a species transfer is not considered well founded. Differences in absorption noted between product compositions when tested in rat skin may not be reproducible in human skin. Particularly acknowledging that factors like lag‐time (usually much higher) and also penetration kinetics may vary significantly between the species. When conducting such an investigation nowadays, it is good practice to either do studies in human skin (from surgery or postmortem) or alternatively in porcine skin, which is much more comparable to human skin than rat [26].

Furthermore, doing the studies with full‐thickness skin but not conducting a full mass‐balance of skin compartments, stratum corneum (understood as the main penetration barrier), epidermis (without stratum corneum), and dermis in addition to the receptor compartment, will not allow to assess whether there is a difference between product composition that is not related to the penetration process but to the distribution profile after the barrier.

### 2.7. Data Treatment

Total amount of drug appeared in the receptor side at time *t* (Q_t_) is calculated by the following equation due to Fick’s second law of diffusion:

(1) Qt=KLC0DtL2−16−2π2∑−1nn2expDn2π2tL2,

where A is the effective diffusion area, K is the partition coefficient of the drug between membrane and vehicle, L signifies the thickness of the membrane, C_0_ represents the drug concentration, which remains constant in the vehicle, and D is the diffusion coefficient.

At steady state, Equation (1) is defined as follows:

(2) QtA=KLC0DtL2−16.

The slope of the linear part of the curve of permeated drug divided by A versus time is defined as flux (J). It should be mentioned that the intercept of the described curve determines the lag time, which is calculated by the following equation:

(3) J=C0 KDL=C0Kp,

where K_P_ is the skin permeability coefficient. The values reported are mean ratios from a minimum of three replicates. J has been used to compare the skin penetration of different formulations. The higher the flux of the drug, the more desirable the formulation is.

### 2.8. Statistical Analysis

All experiments were performed at least in triplicate (*n* = 3), and the results are expressed as mean ± standard deviation (SD). Statistical comparisons between groups were performed using one‐way analysis of variance (ANOVA) followed by Tukey’s post hoc test using GraphPad Prism Version 8.0 (GraphPad Software, USA). A *p* value < 0.05 was considered statistically significant.

## 3. Results

### 3.1. UV and HPLC Analysis

**FIGURE 1:**
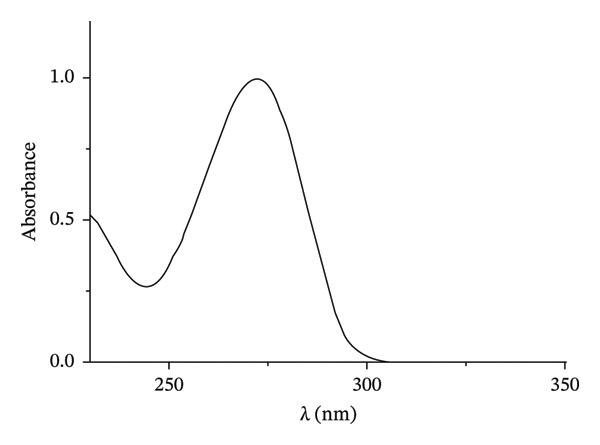
UV absorption spectrum of caffeine solution scanned between 200 and 400 nm. The maximum absorption wavelength (λmax) was observed at 273 nm and was used for HPLC detection [27].

**FIGURE 2:**
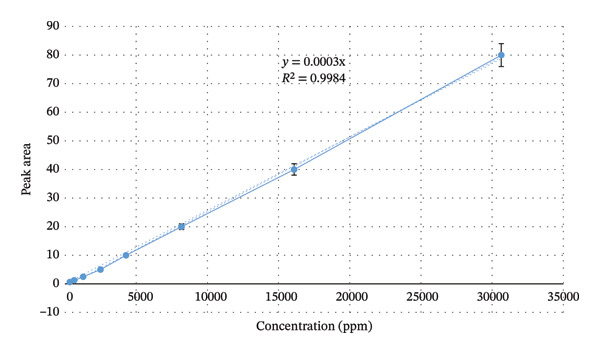
Calibration curve of synthetic caffeine standard solutions analyzed by HPLC‐UV at 273 nm over the concentration range of 0.625–80 ppm. Data represent mean peak area values obtained from triplicate injections.

Based on UV analysis, as shown in Figure 1, 273 nm was defined as the caffeine *λ*_max_ [27]. The calibration curve was prepared by determining the peak area of different concentrations of the synthetic caffeine solution. Then, *C. canephora* extract powder solution was detected by HPLC. Table 1 demonstrates the peak area of each concentration of the synthetic caffeine solution, and the calibration curve is shown in Figure 2.

**TABLE 1:** The HPLC peak area of different concentrations of the synthetic caffeine solution.

Caffeine concentration (ppm)	Peak area
0.625	3447
1.25	6512
2.5	12756
5	24976
10	42879
20	81900
40	160944
80	306478

### 3.2. Preparation Gel of Synthetic/Natural Caffeine Gel

All of the formulations shown in Table 2, except A_3_ and B_3_, were rejected due to the formation of a biphasic gel after 24 h.

**TABLE 2:** The formulation of the gel containing natural or synthetic caffeine.

Formulation	Synthetic caffeine or natural caffeine (g)	Propylene glycol (mL)	Carbopol (g)	Approve or reject
A_1_	0.5	0	0.25	✕
A_2_	0.5	0	1	✕
A_3_	0.5	5	2	✕
A_4_	0.5	10	0.5	✓

### 3.3. Skin Permeation

**FIGURE 3:**
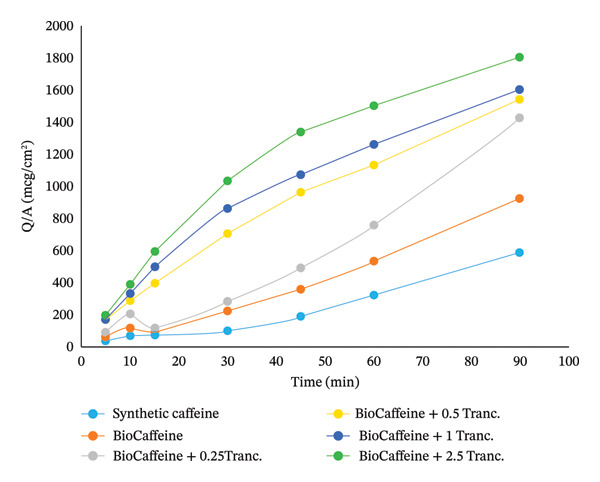
Ex vivo skin permeation profiles of synthetic caffeine gel, natural caffeine gel (*Coffea canephora* extract), and natural caffeine gels containing different concentrations of Transcutol P (0.25%–2.5%) using Franz diffusion cells and rat abdominal skin. Results are expressed as cumulative permeated amount per unit area (Q/A) versus time.

**FIGURE 4:**
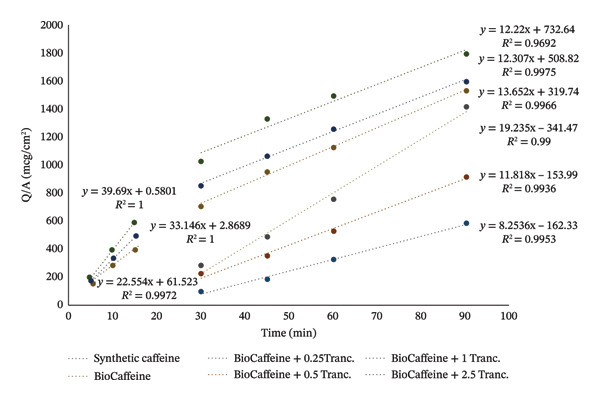
Linear region of the cumulative permeated amount per unit area (Q/A) versus time plot used for determination of caffeine flux through rat skin from different gel formulations.

In this study, the skin penetration of 1% synthetic caffeine gel and 10% natural caffeine gel was measured using a Franz cell. Following baseline comparisons, Transcutol P was incorporated only into natural caffeine gels (0.25%–2.5%) to optimize the higher‐performing formulation, with synthetic caffeine used as a control. Skin permeability was studied in the presence of natural caffeine gel containing 0.25%, 0.5%, 1%, and 2.5% Transcutol P. The results of the studies are given in Tables 3, 4, 5, 6, 7, 8. The permeation profiles of synthetic caffeine, natural caffeine, and natural caffeine in the presence of various amounts of Transcutol P are shown in Figure 3. Furthermore, the linear part of the curve of Q divided by A versus time was used to determine and compare the fluxes in the prepared formulations (Table 9 and Figure 4). These results indicated that the skin permeation of the natural caffeine gel was higher than that of the synthetic caffeine gel. Additionally, permeation was improved by increasing the amount of Transcutol in the formulation of the natural caffeine gel.

**TABLE 3:** The skin penetration of 1% synthetic caffeine gel.

Time (min)	Q/A norm
5	37.58 ± 0.42
10	72.09 ± 0.53
15	74.23 ± 0.70
30	101.24 ± 0.75
45	192.82 ± 0.98
60	325.37 ± 0.95
90	588.32 ± 0.86

**TABLE 4:** The skin penetration of 10% natural caffeine gel.

Time (min)	Q/A norm
5	61.89 ± 0.31
10	122.66 ± 0.94
15	95.56 ± 0.91
30	226.21 ± 0.81
45	358.06 ± 0.37
60	533.42 ± 0.72
90	925.41 ± 0.81

**TABLE 5:** The skin penetration of natural caffeine gel containing 0.25% Transcutol.

Time (min)	Q/A norm
5	93.80 ± 061
10	204.53 ± 0.8
15	121.20 ± 0.91
30	285.60 ± 0.84
45	492.10 ± 0.33
60	760.67 ± 0.91
90	1423.72 ± 1.09

**TABLE 6:** The skin penetration of natural caffeine gel containing 0.5% Transcutol.

Time (min)	Q/A norm
5	170.85 ± 0.63
10	293.95 ± 0.39
15	396.38 ± 0.13
30	709.95 ± 0.83
45	962.97 ± 0.21
60	1134.19 ± 0.38
90	1543.50 ± 0.4

**TABLE 7:** The skin penetration of natural caffeine gel containing 1% Transcutol.

Time (min)	Q/A norm
5	168.42 ± 0.79
10	334.68 ± 0.84
15	499.88 ± 2.02
30	862.08 ± 0.44
45	1073.17 ± 0.96
60	1263.41 ± 0.64
90	1605.78 ± 0.59

**TABLE 8:** The skin penetration of natural caffeine gel containing 2.5% Transcutol.

Time (min)	Q/A norm
5	198.23 ± 0.88
10	399.09 ± 0.85
15	595.13 ± 0.91
30	1038.29 ± 0.29
45	1339.04 ± 0.56
60	1502.91 ± 0.62
90	1799.73 ± 0.67

**TABLE 9:** Flux, Q_30_, Q_90_, and *R* ^2^ value in the prepared formulations.

Formulation	Flux 1 (μg/cm^2^ h)	Flux 2 (μg/cm^2^h)	*R* ^2^	*R* ^2^	Q_30_	Q_90_
Synthetic caffeine gel	—	8.25		0.99	101.24	588.32
Bio caffeine gel	—	11.81		0.99	226.21	925.41
Bio caffeine gel (Transcutol P 0.25%)	—	19.23		0.99	285.6	1423.72
Bio caffeine gel (Transcutol P 0. 5%)	22.55	13.65	0.99	0.99	709.95	1543.5
Bio caffeine gel (Transcutol P 1%)	33.14	12.30	1	0.99	862.08	1605.78
Bio caffeine gel (Transcutol P 2.5%)	39.69	12.22	1	0.96	1.29038	1799.73

*Note:* Flux 1 (the intercept of the initial linear part of the Q/A vs. time plot). Flux 2 (the intercept of the initial linear part of the Q/A vs. time plot).

## 4. Discussion

**FIGURE 5:**
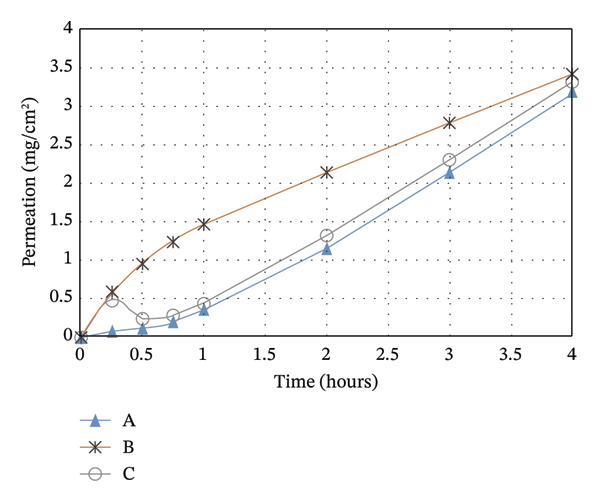
Schematic representation of three proposed skin permeation patterns of topical drugs: (A) classical permeation model with positive lag time, (B) burst permeation pattern, and (C) burst permeation followed by a secondary steady‐state phase [31–32].

Caffeine has been used in cosmetic and skin products for a long time due to its many promising properties, such as anticellulite and antiaging effects [28]. It is preferred to utilize topical caffeine for cellulite treatment in order to escape its undesirable adverse effects, such as nervous excitation, sleep disturbances, heart rate increase, and diuresis [29]. To avoid these effects, it is preferred to improve the skin permeation of caffeine in order to deliver and dissolve it into subcutaneous fat [30]. In this research, Transcutol P was used for this purpose. To investigate the release pattern of caffeine from the gel formulation, a graph was plotted showing the amount of drug passing through the unit of skin surface over time. Previous studies have suggested different patterns for drug penetration through the skin. Three of these patterns are schematically shown in Figure 5 [31–32]. In the classical model of skin transportation of drugs (Diagram A), the amount of drug absorption per unit of skin surface is initially low and does not follow a linear pattern. As time passes, the amount of penetration increases and takes on a linear trend. In this transportation model, the lag time of the drug, which is obtained by extending the linear part of the graph and intersecting with the time axis, is positive. This means that in the classic model of skin penetration of drugs, the lag time is considered an inherent characteristic. This is because, at first, the skin is not saturated with the drug. Thus, the drug dissolves in the skin fats, and the amount of absorbed drug is low. After the skin is saturated with the drug, the process of absorption and passage of the drug reaches a steady state, and the absorption graph becomes almost linear. This is a major challenge for the classic model because most skin products have a short residence time on the skin. The drug can be removed from the skin by perspiration, washing, and cleansers. Therefore, drug transportation in the early stages is more important, especially for products with a short residence time, such as aqueous and hydro alcoholic solutions, gels, or lotions [31]. In the second type of pattern (Diagram B), the slope of the first part is steeper than the linear and steady‐state part. This type of drug penetration pattern, known as burst transportation, occurs mostly when high‐efficiency permeation enhancers are used in the formulation. In this case, before the skin is saturated with the drug, the permeation enhancer transports the drug into the skin along with it (cotransportation). Since the permeation enhancer used in the formulation tends to quickly penetrate the skin, this effect is more noticeable in the initial times. With increasing time and decreasing amounts of permeation enhancer, this effect decreases until it reaches a steady state [32]. In the third type of pattern (Diagram C), like the burst penetration pattern, the drug is initially absorbed in the skin at a high amount, but its concentration decreases again in the receptor phase of cell diffusion. After a certain period of time, it starts to increase again and reaches a linear or steady state. In other words, a peak in the concentration of the drug is observed in the initial times. Based on previous studies, this pattern is observed in formulations with appropriate permeation enhancers and very lipophilic drugs. First, the enhancer transports the drug through the skin (before the skin is saturated with the drug). Then, due to the lipophilic nature of the drug, it dissolves in the subcutaneous fats over time, and its concentration decreases in the receptor phase. This decreasing process continues until the skin fats are saturated with the drug, and after the skin is saturated, the drug concentration in the receptor phase increases again and approaches a steady state [32].

As shown in Figure 3, the pattern of skin penetration in the initial formulation containing synthetic caffeine follows the classic pattern. However, the next two formulations (the natural caffeine formulation and natural caffeine + 0.25% Transcutol formulation) follow the third pattern. In the other three formulations, which contain natural caffeine along with 0.5%, 1%, and 2.5% Transcutol P, the burst permeation pattern was seen. In the burst pattern, two phases are seen in the Q/A versus time plot. The first phase is a result of the burst transportation of the drug. While the second phase is associated with the steady state condition of the drug permeation through the skin. Furthermore, a direct correlation was seen between the amounts of penetrated caffeine and the concentrations of the Transcutol P.

The flux is not important for drugs that follow a classic pattern of passing through the skin. This is because the drug may be removed from the skin before reaching the linear part of the plot due to its low residence time. However, for drugs that follow a burst transportation pattern, the flux is important. This is because the drug starts to pass through the skin as soon as it is placed on the site. The initial part of the Q/A plot (Flux 1) demonstrates the rate of drug permeation through 1 cm^2^ of skin surface during the initial use of the drug. The slope of the initial linear portion of the Q/A versus time plot (Flux 1) is important in the burst release pattern, as it shows the rate at which the drug passes through the skin when it is placed on the site. The slope of the final linear portion of the Q/A versus time plot (Flux 2) in the burst pattern indicates the final balance of the drug on the skin. Mustafa et al. prepared a gel formulation of caffeine and studied its permeation using a Franz cell and human skin. The maximum flux was found to be 4.78 μg/cm^2^ min (effect of concentration on skin permeation of caffeine from gel formulations). According to Table 8, the highest Flux 1 is seen in the natural caffeine gel formulation with 2.5% Transcutol P (39.69 μg/cm^2^min). Additionally, the flux in the natural caffeine formulations is higher than that in the synthetic caffeine formulation. Considering the point that drug concentration, skin surface, and the amount of product used on the skin are the same in all formulations, it can be concluded that the increase in the flux is related to the use of natural caffeine instead of synthetic caffeine and the use of a permeation enhancer.

It is important to address the limitations of the present study, particularly regarding the skin model used. The permeation experiments were conducted using full‐thickness rat skin, which is known to be more permeable than human skin due to structural differences, including a thinner stratum corneum and a higher density of hair follicles [24, 25]. Consequently, the absolute penetration values and kinetics observed here, such as a potentially shorter lag time, may not be directly translatable to the human situation. The relative differences in permeation between the synthetic and natural caffeine formulations in this model, however, provide valuable preliminary data on their comparative behavior. Furthermore, the use of full‐thickness skin without a detailed mass balance analysis across skin compartments (stratum corneum, viable epidermis, and dermis) limits our ability to discern the precise distribution profile of caffeine after penetration. Therefore, these results should be interpreted as a foundational step. Future work will necessitate the use of more representative skin models, such as human or porcine skin [26], coupled with a comprehensive tape‐stripping and compartmental analysis to fully validate these findings and better predict human topical application.

Furthermore, the application dose used in this in vitro study (∼263 mg gel/cm^2^) was selected to maintain infinite dose conditions, ensuring a constant concentration gradient for a standardized comparison of the formulations. It is important to note that this dose is substantially higher than typical consumer use (e.g., ∼1–5 mg cream/cm^2^). Consequently, while this model effectively reveals differences in the permeation potential between the synthetic and natural caffeine gels, the absolute amounts and rates of permeation reported here are specific to these experimental conditions and should not be directly equated to expected in vivo bioavailability.

It should be noted that the penetration pattern of caffeine in all of the formulations follows the same pattern for the second and third types. Thus, using the lag time parameter to compare the effects of the formulations is not suitable. The uniqueness of this study lies in demonstrating superior flux from natural caffeine gels augmented by Transcutol P, surpassing reported values for synthetic caffeine alone, with implications for ecofriendly anticellulite therapies.

## 5. Conclusion

Based on the comparison of flux values between different formulations, it can be concluded that the addition of 2.5% Transcutol P to the formulation containing *C. canephora* extract resulted in the highest caffeine flux. Therefore, the optimal formulation identified in this study was the natural caffeine gel containing 2.5% Transcutol P.

Nevertheless, several limitations should be acknowledged. The permeation studies were conducted using rat skin, which is more permeable than human skin and may not fully predict human topical absorption behavior. In addition, a detailed compartmental distribution analysis within the skin layers was not performed. Future studies should therefore validate these findings using human or porcine skin models and investigate long‐term skin retention and in vivo performance to better assess the translational potential of the proposed formulations.

## Author Contributions

Nastaran Seyyed Abbasi performed the research and writing–original draft. Fatemeh Soltanmohammadi prepared the tables and writing–original draft. Mohammad Charkhpour conceptualized and designed the study and also analyzed the data. Javad Shokri contributed to the conceptualization and writing–review and editing of the manuscript.

## Funding

The current research was funded and granted by Tabriz university of Medical Sciences (grant number: 1400.083).

## Disclosure

All authors have read the journal’s authorship agreement, and the manuscript has been reviewed and approved by all named authors.

## Ethics Statement

The experiments were performed in accordance with the ethical committee of the Tabriz University of Medical Sciences (IR.TBZMED.VCR.REC.1400.083).

## Conflicts of Interest

The authors declare no conflicts of interest.

## Data Availability

The data that support the findings of this study are available from the corresponding author upon reasonable request.
